# Students’ knowledge of and conservation attitude toward the black-necked crane (*Grus nigricollis*) in Guizhou, China: insights for conservation

**DOI:** 10.1186/s13002-022-00536-6

**Published:** 2022-05-09

**Authors:** Yun Ruan, Yalong Li, Yuanping Xia, Tailin Yu, Chuanyin Dai

**Affiliations:** 1 Key Laboratory of Ecology of Rare and Endangered Species and Environmental Protection (Guangxi Normal University), Ministry of Education, 1 Yanzhong Road, Guilin, 541006 China; 2 Guangxi Key Laboratory of Rare and Endangered Animal Ecology, Guangxi Normal University, Guilin, 541006 China; 3 Caohai Ecological Station, Guizhou Academy of Science, Weining, 553100 China

**Keywords:** Black-necked crane, Junior student, Ecosystem service, Attitude, Knowledge, Human–bird relationship

## Abstract

**Background:**

The persistence of threatened and protected wildlife depends not only on habitat suitability but also remarkably on local communities’ acceptance. The black-necked crane (*Grus nigricollis*) is a flagship species for conservation on the plateau in western China. However, the human dimension has been completely ignored in the decision-making process for conservation.

**Methods:**

In this study, a questionnaire survey aiming to assess knowledge of and conservation attitude toward this bird was carried out among 1042 students of 7th and 9th grade from 12 schools in Weining county, Guizhou province, which has a large wintering population in an urban wetland. Logistic regression was used in the generalized linear model to identify the determinants that significantly affect students’ knowledge of and conservation attitude toward this species.

**Results:**

Most students have positive attitudes toward conservation, which is significantly affected by awareness, knowledge of this bird and grade. However, they have somewhat limited knowledge of this bird’s biology and ecosystem services (nature’s contributions to people). Knowledge was found to link with observation of the bird and grade, while observation related to the experience of visiting the wetland. Social media is the most cited resource to obtain knowledge on this bird.

**Conclusion:**

It is suggested that local conservation experts could help introduce more information on the black-necked crane in the schools and help conduct outdoor education activities in and around the wetland. Traditional knowledge and culture could also be incorporated into the conservation awareness enhancing program. This study focuses on the human dimension for conserving the black-necked crane in China, showing significant implications in the design and application of effective measurements to improve students’ perception and attitude toward its conservation. Future assessments should include other local populations, such as farmers, fishers, and urban citizens.

## Introduction

The current loss of biodiversity requires various effective measures to conserve wildlife and associated ecosystems, such as law enforcement, protecting areas, ecosystem restoration, and invasive species management [1, 2]. However, the coexistence of protected wildlife with local people depends not only on habitat suitability but also on local communities’ attitude and acceptance [3–6]. This is because conservation may bring additional burdens and affect local populations’ livelihoods. For example, protected species can cause livestock and crop loss [7], or large carnivore species can threaten the lives of others [8]. Additionally, it requires local people to observe and sometimes positively participate in protection activities. As such, local support is one of the key factors to enable the long-lasting success of wildlife conservation [9, 10]. On the other hand, local people might have a prejudiced perception against a protected species or the use of indispensable natural resources being restricted due to conservation implementations. In such cases, local people may be unwilling to engage in conservation actions even if they supposedly claim to support the protection program [9]. Therefore, human dimensions of wildlife conservation, such as assessing perceptions of and attitudes toward wildlife and conservation, should be considered and have already emerged as a paramount aspect of studies in wildlife conservation [11, 12].

In this regard, several studies demonstrated that local populations’ conservation attitude toward a species might have a link with the level of knowledge they hold about that species [13, 14]. It can enable the local people to have a better and more comprehensive appreciation for the species and enjoy it [14]. Consequently, knowledge of and attitude toward a wide range of animals have been investigated among the local populations in different regions across the world, such as wild boars (*Sus scrofa* Linnaeus, 1758) [15], brown bears (*Ursus arctos* Linnaeus, 1758) [6], wolves (*Canis lupus* Linnaeus, 1758) [16, 17], bats [18] and bees [19]. On the other hand, the concept of ecosystem service (or nature’s contributions to people [20]) has attracted extensive attention in conservation in the past decades [21]. It can be defined as a full range of benefits that humans can obtain directly or indirectly from ecosystems generated by various ecological processes and ecosystem properties [20, 22, 23]. The ecosystem service approach is a valuable way to convey society’s dependence on the various natural ecosystems [22, 24, 25]. Identifying and quantifying the value of the services help muster support for biodiversity protection and influence public attitude and policy decision [26], making the evaluation of ecosystem service the focal topic and mainstream in recent years. Within this context, the social-cultural valuing approach has many advantages over the traditional economic and ecological approaches [27, 28]. For example, it can be used to detect the services that local people have realized or to identify the most important service to them, as it applies research methods from the social sciences (e.g., interviews) and values ecosystem services in non-monetary terms [28, 29].

The black-necked crane (*Grus nigricollis* Przhevalsky, 1876) is a large wading bird in the order of Gruiformes, which is the only crane that lives on the plateau all its life [30]. At present, its population has been estimated at appropriately 10,000 individuals in total. Despite a slowly increasing population trend, it was identified as a global vulnerable (VU) species by the International Union for Conservation of Nature (IUCN) and listed as a Class I national key protected wildlife in China. This bird breeds in the Qinghai-Tibetan Plateau, with a small population in India. It winters in the lower elevations of the Qinghai-Tibetan and the Yunnan-Guizhou Plateaus, with a few occurring in Bhutan and northeast India [30]. To conserve this bird, most of its major breeding and wintering areas have been designated as protected areas in China. At the same time, scientific studies have been intensive and gradually increased in recent years. However, most studies were concentrated in the field of biology or ecology [31–35] and investigated topics such as the diurnal time budget during the breeding season [35] or the migration route and stopover sites [33]. To our knowledge, there are thus still limited studies focusing on human dimensions, especially on the assessment of public knowledge of and conservation attitude toward the black-necked crane.

To fill this knowledge gap, a survey was conducted to assess secondary school students’ knowledge of and attitudes toward the black-necked crane in Weining county, Guizhou province, China. A large wintering population occurs here in an urban freshwater wetland. Studies have emphasized that the development of students’ environmental knowledge, ecological understandings, and environmental attitudes and behaviors are essential because today’s students will be responsible for nature conservation in the future [36, 37]. However, if negative attitudes are not removed or changed, they may cause negative rather than positive behaviors for wildlife conservation in adults [13, 19]. On the other hand, unveiling the factors influencing certain attitudes is important for designing an appropriate education program to raise the students’ awareness of wildlife conservation [38, 39].

In this study, a survey was carried out among the 7th and 9th grade students from 12 junior middle schools to assess their attitudes toward and knowledge about the biology and ecosystem services of the black-necked crane. The main purposes of this study were 1) to investigate their knowledge of and attitude toward the black-necked crane; 2) to identify how sociodemographic factors related to knowledge and attitude; 3) and based on these findings, to design an effective and feasible strategy to shape a more favorable attitude in students toward the conservation of the black-necked crane.

## Materials and methods

### Study area

**Fig. 1:**
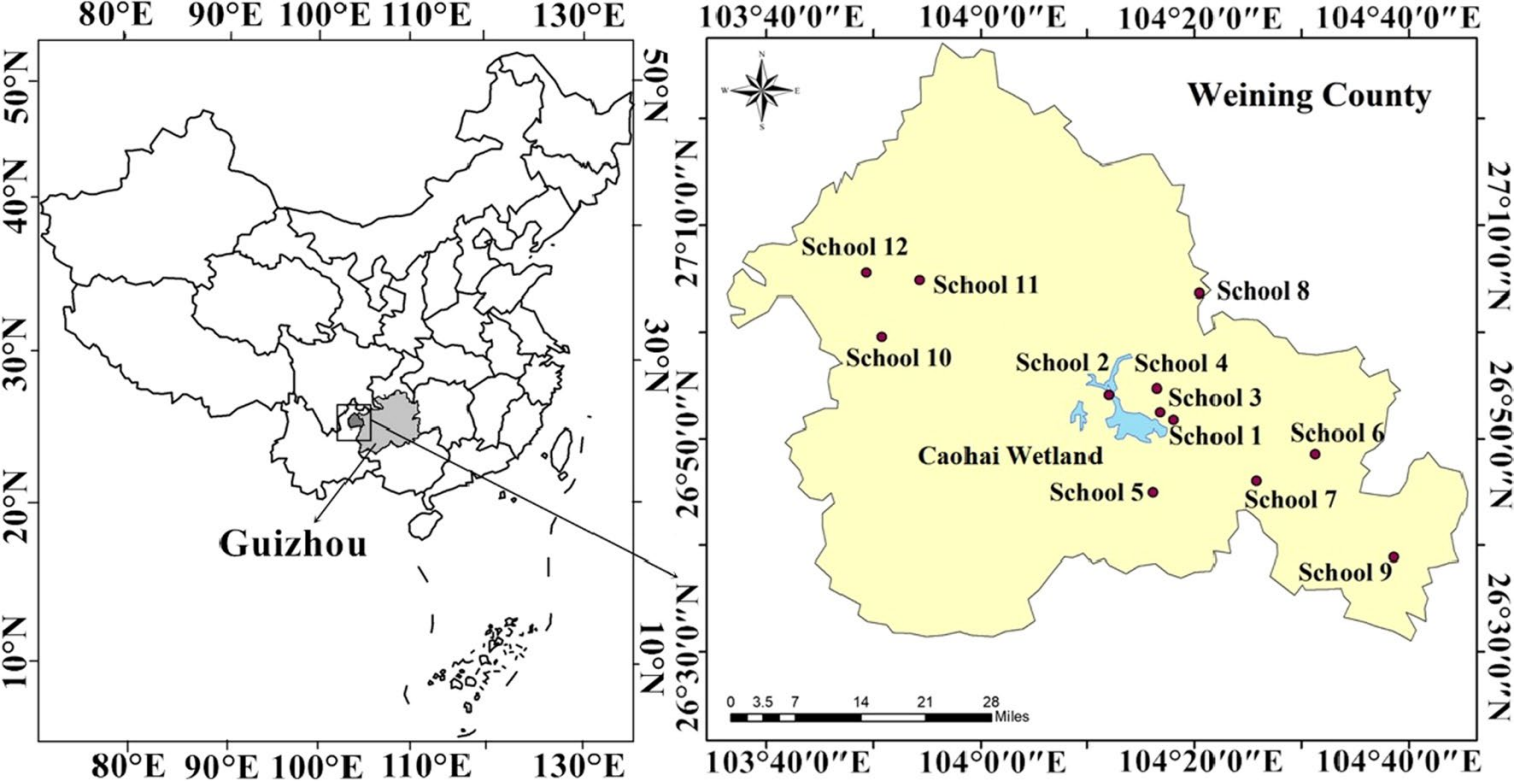
Map showing the study area and the interviewed schools

This study was conducted in Weining county, northwest Guizhou province in China (Fig. 1). The county has a population of approximately 1.3 million, of which 25% are ethnic, cultural groups, such as Mongolian, Gelao, Tujia, Bai, Yi, Hui, Miao and Buyi. Agriculture and migrant labor service are the main avenues for the local farmers. With an average altitude of 2200 m, the climate is subtropical monsoon humid. It is cool in summer but warm in winter, except on rainy and snowy days. Due to the large population of the black-necked crane wintering in an urban lake, the Caohai wetland (approximately 25 km^2^), a national natural reserve (approximately 120 km^2^), was set up years ago and has been identified as a critical first-class wetland by the China Biodiversity Conservation Action Plan [40]. Additionally, population breeding in the eastern Qinghai-Tibetan Plateau annually overwinters in this wetland, with up to 2000 individuals [30]. Moreover, 80% of the cranes forage in the farming areas around the wetland, mainly feeding on potatoes, corn, and other residual crops [31] and return to the wetland for resting at night [41]. Besides this flagship black-necked crane species, the wetland provides a habitat for nearly 100,000 waterbirds every winter, including many rare and beautiful species, such as common cranes *Grus grus* (Linnaeus, 1758), whooper swans *Cygnus cygnus* (Linnaeus, 1758), and tundra swans *Cygnus columbianus* (Ord, 1815). Furthermore, the urban region of the county is built adjacent to the eastern and northern borders of the reserve. Due to the greater conveniences for accommodation and traffic, the Caohai wetland has attracted many bird-watchers during the wintering periods, making it a significant economic, symbolic, and social area for the county [40].

### Data collection: questionnaire survey

A semi-closed questionnaire survey was conducted among grade 7th and 9th students in 12 middle schools from October 2020 to April 2021. The locations of these schools are shown in Fig. 1. The questionnaires first asked the students to list the three most famous things in their hometown or county. Then, it collected students’ demographic information, such as age, gender, ethnicity, and family location (answering options were urban region of the county, town, rural area surrounding the Caohai wetland, and other rural areas). Fifteen simple questions were formulated that the students could easily understand. The questions generally covered five aspects.

One question was used to detect students’ awareness of the black-necked crane: 1) Do you know the black-necked crane? Then, three other questions were used to identify their knowledge sources. So, if they answered yes in question 1, the following questions were (2) Have your parents or family members told you something about the black-necked crane? (3) Have your teachers told you something about the black-necked crane? (4) Have you read an introduction to the black-necked crane from a poster, TV, newspaper, or website? The answering options for the four questions were “Yes” and “No” and were coded as 1 and 0, respectively.

Three questions were used to investigate students’ experience with the black-necked crane and the Caohai wetland: (1) Have you seen the black-necked crane? The answering options were again “Yes” and “No” and coded as 1 and 0, respectively. (2) If Yes, where did you see it? Students could write the locations on the questionnaire. (3) Have you been to the Caohai wetland or nearby? The answering options were again “Yes” and “No” and coded as 1 and 0, respectively.

Five questions were used to assess students’ knowledge of the black-necked crane’s biology. (1) What is the black-necked crane? Birds, reptiles, mammals or amphibians? The option of “I don’t know” was also provided. One point was rewarded if the student selected the correct answer (birds). (2) Is the black-necked crane a national critical protected animal? (3) Do you agree that only Weining county has the black-necked crane in Guizhou? (4) Do you agree that the black-necked crane usually appears in Weining from November to March of the following year? (5) Do you agree that the black-necked crane may eat farmers’ crops? For questions 2 to 5, the answering options were “Yes,” “No,” and “I’m not sure.” One point was rewarded with the answer of “Yes.” Composite scores were used in the statistical analyses.

One simple question was used to record students’ attitudes toward conserving the black-necked crane: Do you think the black-necked crane should be protected? Answering options were “Yes,” “No,” and “I’m not sure.” “Yes” was coded as 1, while “No” and “I’m not sure” were coded as 0.

The last question was open-ended and was used to identify the students’ perception of the ecosystem services provided by the black-necked crane: What benefits do you think the black-necked crane can provide to the local people? They were asked to write the answers on the questionnaire, and “I don’t know” was allowed. The services mentioned by the students were classified according to the standard classification outlined by the Millennium Ecosystem Assessment [23] and Green and Elmberg [26]. One point was awarded for each service listed by the students, and the composite scores were used in the statistical analyses.

The questionnaire provided to the students was in Chinese. Such studies do not require the approval of an ethics committee or similar body under Chinese regulations. A particular date and a convenient time were chosen for carrying out the survey. On the scheduled date and time, students were retained in their classrooms, and questionnaire sheets were distributed after a brief explanation of the aim of the investigation given by their teachers or the author C.D. During the data collection steps, complete anonymity was guaranteed to the participants. They also had the option to decline to fill in the questionnaire by presenting a blank questionnaire back. One class from 7 and 9th grade was selected in each of the 12 schools, while 50 questionnaires were distributed in each class with approximately 60 students.

### Data analysis

All the quantitative and qualitative data were coded and entered using MS Excel. All the data analyses were conducted in R [42]. Descriptive statistics, such as average and proportion or frequency, were analyzed for the corresponding variables. Spearman’s product-moment correlation coefficient was used for exploring the relationship among variables.

Logistic regression was used in the generalized linear model to identify the determinants that significantly affect students’ conservation attitude and awareness of and experience with the black-necked crane. Scores on biological knowledge and ecosystem services were combined when exploring the influencing factors for conservation attitude. In addition, overdispersion for the dependent variable was detected to determine the appropriate distribution family type for a fitting model.

Although students’ biological knowledge score conforms to the normal distribution, a linear relationship with each independent variable was not strongly supported as indicated by the Kolmogorov–Smirnov test (P < 0.05) and parallel line test (P < 0.05). Therefore, this variable was divided into two categories. Those with scores of 0 to 2 were classified into a lower group and those with scores of 3 to 5 into a higher group. This way, the variables that significantly impact students’ biological knowledge scores could be identified by binary logistic regression analysis.

## Results

### Characteristics of the students

Of the distributed questionnaires, 133 were completely blank. In addition, 25 students claimed that they did not know the black-necked crane but presented the knowledge source. This indicated that they did not understand the questionnaire well or just answered carelessly. All these questionnaires were excluded in the following analyses. Among the 1042 respondents, 46.93% (n = 489) were male, while 53.07% (n = 553) were female. The number of 7th and 9th grade students was 482 (46.26%) and 560 (53.74%), respectively. The age of students ranged from 11 to 20, with an average of 14.23. 70.92% of the students were from rural areas (n = 739), whereas most were Han population (n = 849, 81.48%).

### Knowledge source

A total of 954 students (91.55%) indicated that they knew the black-necked crane. Social media (n = 862; 82.73%) is the most cited source for the students to appreciate the black-necked crane, followed by the school education (n = 769; 73.80%) and family members (n = 591; 56.72%). A small number of respondents claimed they obtained information from other sources, such as their friends and classmates (n = 29; 2.78%). Awareness on the black-necked crane was correlated with grade (r = 0.27; P < 0.001). Logistic regression analyses showed that only grade has a significant effect (Table 1; *β* = 1.18; P < 0.001). Among the 88 (8.45%) students who declared that they did not know the black-necked crane, most (n = 79) were in 7th grade.

**Table 1:** Logistic regression analyses identified grade as the only significant factor to impact the students’ awareness

	Β	SE	*z* value	Pr ( >|*z*|)
Intercept	− 6.11	1.85	− 3.31	0.0009
School	− 0.05	0.05	− 0.99	0.32
Grade	1.23	0.24	5.18	2.24e−07
Age	− 0.06	0.16	− 0.36	0.72
Gender	0.22	0.25	0.91	0.36
Ethnicity	0.18	0.31	0.57	0.57
Address	− 0.10	0.17	− 0.56	0.57
observation	18.33	800.50	0.02	0.98
Visiting experience	− 0.06	0.26	− 0.24	0.81

### Direct experience

65.74% (n = 685) of the students reported visiting the Caohai wetland, while 40.50% (n = 422) have observed the black-necked crane. Three hundred thirty-five students (32.15%) claimed that they had no experience with both the black-necked crane and the Caohai wetland. The opportunity to see the black-necked crane was positively related to the visiting times to the Caohai wetland (r = 0.5; P < 0.001) but negatively affected by the school distance to the Caohai wetland (r = − 0.41; P < 0.01). Logistic analyses (Table 2) showed that the chance to see the black-necked crane is 14.27 times higher for students who have been to the wetland than those who have not (Exp.coef = 14.27; P < 0.001). However, visiting the wetland is negatively related to the students’ family locations (r = − 0.35; P < 0.01) and their attending schools (r = − 0.36, P < 0.01), but positively related to grade (r = 0.11; P < 0.01). Logistic analyses (Table 3) showed that family location is the most influencing factor that determined the students’ experience with the wetland (*β* = − 0.82; P < 0.001). The closer the school (*β* = − 0.18, P < 0.001) and family location to the Caohai, the more chances students have to visit the wetland and the more experiences they have with the black-necked crane. The odds ratio between 9 and 7th grade is 1.25, indicating students in 9th grade have more chances to visit the wetland than those in 7th grade (Exp.coef = 1.25).

**Table 2:** Analyses of the determinants that impacted the students’ observation of the black-necked crane

	Β	SE	z value	Pr ( >|z|)
Intercept	− 4.02	0.99	− 4.06	4.92e−05
School	− 0.21	0.03	− 7.89	3.01e−15
Grade	0.18	0.13	1.44	0.15
Age	0.09	0.10	0.95	0.35
Gender	− 0.09	0.16	− 0.60	0.55
Ethnicity	− 0.20	0.21	− 0.99	0.32
Address	0.01	0.07	0.14	0.89
Visiting experience	2.66	0.24	10.97	< 2e−16

**Table 3:** Variables that significantly impacted the students’ experience with the Caohai wetland

	Β	SE	z value	Pr ( >|z|)
Intercept	3.36	0.98	3.43	0.0006
School	− 0.19	0.02	− 7.69	1.51e−14
Grade	0.28	0.11	2.49	0.01
Age	− 0.06	0.09	− 0.66	0.51
Gender	− 0.15	0.15	− 1.00	0.32
Ethnicity	− 0.28	0.20	− 1.38	0.17
Address	− 0.81	0.12	− 6.79	1.16e−11

### Biological knowledge

Overall, the students had rather limited knowledge of the biology of the black-necked crane as the average score obtained was only 2.21 (Full mark = 5). The proportions for correctly answering the five questions were 83.59%, 76.87%, 16.6%, 37.14% and 6.33%, respectively. 7.49% (n = 78) of the students selected the wrong options for all questions. 14.40% (n = 150) only obtained a composite score of 1. The highest proportion of the students (40.88%; n = 426) fetched 2 points. 25.91% (n = 270) and 9.98% (n = 104) scored 3 and 4, respectively. Only 1.34% (n = 14) had a full mark, answering all the questions correctly.

School number two, closest to the Caohai wetland, obtained the highest average score of 2.85, while the farthest school (number twelve) had the lowest score of 1.57 (F = 48.95; P < 0.001). The average score of 2.58 for the students in 9th grade was significantly higher than those in 7th, with an average score of 2.02 (t = − 11.54; P < 0.001). Students living in the rural region around the Caohai wetland (2.89) have more biological knowledge than those in the other three regions (Weining county = 2.54; Towns = 2.38; Other rural areas = 2.25, F = 24.51; P < 0.001). Furthermore, the students who have observed the black-necked crane (2.61) fetched higher scores than their counterparts (2.13; t = − 10.59; P < 0.001). This is also the case for the students visiting the Caohai wetland (Visited = 2.48; Not visited = 2.06; t = − 7.44; P < 0.001). However, no significant differences have been observed among the other two variables (Male = 2.39; Female = 2.31; t = 0.60, P = 0.55; Ethnicity: Han = 2.31; Other ethnicities = 2.49; t = 1.61; P = 0.11).

It should be mentioned that among the 88 students who responded that they did not know the black-necked crane, 36 students still got 1 point, while nine students got 2 points and three students got 3 points. The three students with a score of 3 were classified into the lower group in the logistic regression analyses since this is irrational. Logistic regression analyses identified that school (*β* = − 0.06; P < 0.01), grade (*β* = 0.64; P < 0.001), ethnicity (*β* = − 0.5; P < 0.01), and observation (*β* = 0.71; P < 0.001) have significant impacts on students’ biological knowledge score (Table 4). The odds ratio between students who have seen the black-necked crane and those who have not is 2.10 (Exp.coef = 2.10), while students in 9th grade are better than those in 7th grade (Exp.coef = 1.95).

**Table 4:** Variables that significantly impacted students’ biological knowledge

	Β	SE	z value	Pr ( >|z|)
Intercept	− 5.76	0.88	− 6.57	5.01e−11
School	− 0.06	0.02	− 2.62	0.01
Grade	0.64	0.11	5.71	1.14e−08
Age	0.03	0.09	0.39	0.70
Gender	− 0.15	0.14	− 1.04	0.30
Ethnicity	− 0.50	0.18	− 2.78	0.005
Address	0.05	0.07	0.76	0.45
Observation	0.71	0.17	4.21	2.56e−05
Visiting experience	0.10	0.18	0.55	0.59

### Perception of ecosystem services

**Fig. 2:**
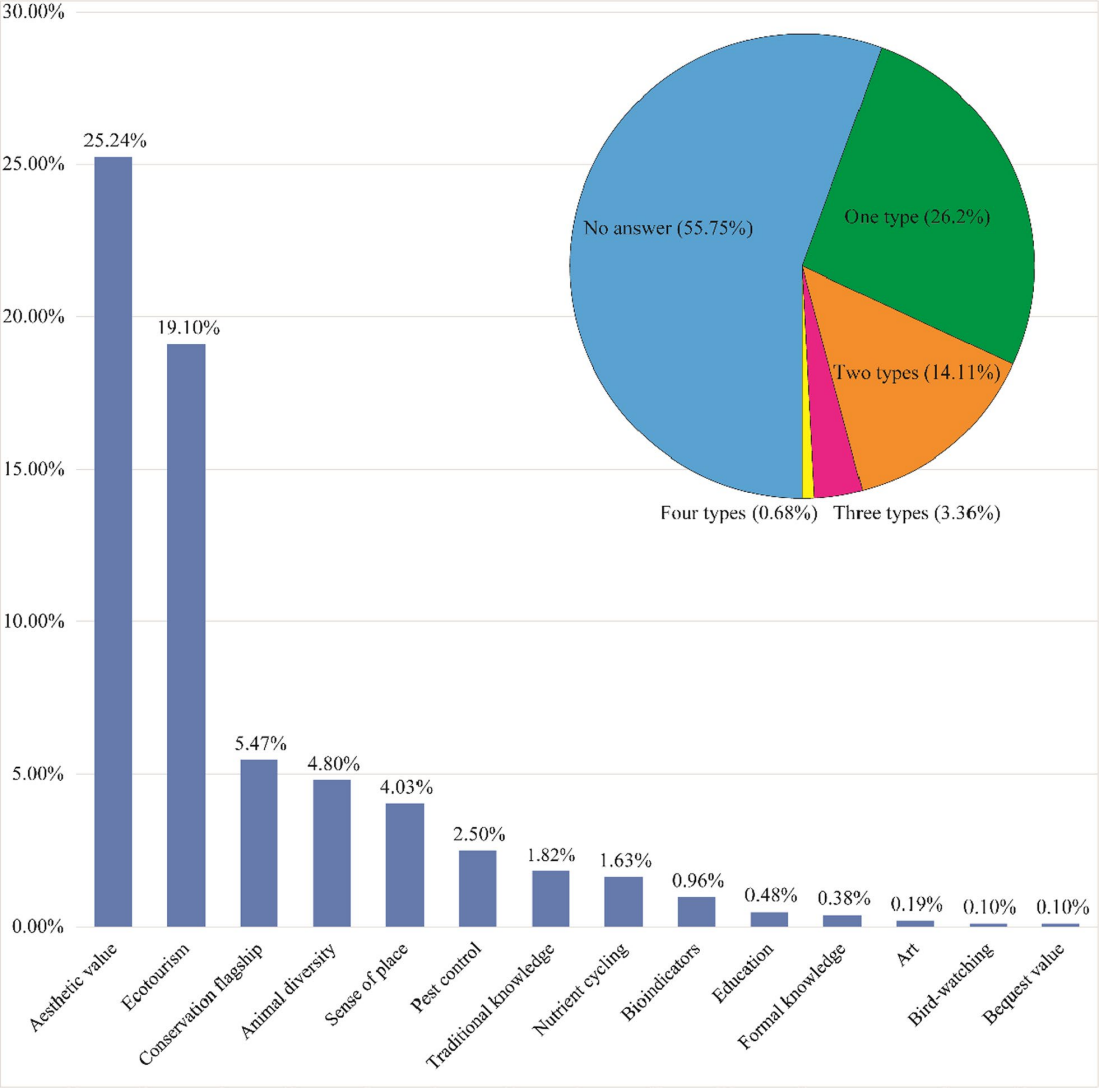
Ecosystem services mentioned by the students. Pie chart shows the percentages for the number of ecosystem services mentioned by each student. Bar chart indicates the percentages for each ecosystem service mentioned by the students

Overall, only 44.25% (n = 461; Grade 7: n = 116; Grade 9: n = 345) of the students presented the ecosystem services provided by the black-necked crane, with an average score of 0.67 (Fig. 2). A total of fourteen services were reported (Table 5). However, at most, four types were cited by one student (n = 6; 0.58%). Additionally, most students can report only one type of service (n = 273, 26.20%). Esthetic value (n = 263; 25.24%) and ecotourism (n = 199, 19.10%) have relatively higher references (Fig. 2). Both bird-watching and bequest values were cited only by one student. It should be mentioned that 26 (2.50%) students thought that the bird could help catch pest insects for local farmers.

**Table 5:** Students’ perception of services provided by the black-necked crane

Categories	Ecosystem services	Narrative examples
Supporting	Nutrient cycling	Droppings can bring nourishment to the land
		The bird can loosen farmers’ fields in the process of foraging
	Animal diversity	The bird’s arrival enriches the animal species in Weining and maintains the balance of the Caohai ecosystem
	Bioindicator	Studying the behavior and lives of migratory like the black-necked crane can help to study how to protect the habitat of birds and reflect and test the ecological environment from many angles
Regulating	Pest control	The bird eats pest insects
Cultural	Ecotourism	Promote the development of tourism in Weining
		Promote locals to establish a reserve and ecological park, thus promoting sustained economic benefits and development of Guizhou and improving the quality of life of the local people
	Sense of place	The bird has promoted the fame of Weining
	Bird-watching	Meet the needs of bird-watchers
	Art	To provide inspiration and material for writers, for example, articles about the black-necked crane
	Esthetic value	Watching the bird in nature allows people to appreciate its beauty, feel physically and mentally happy, and will lighten their mood
		It adds to the beauty of the landscape and living environment of residents in the area of Caohai
		Their flying shape and figure in the sky are very beautiful, often accompany the students, and enrich the scenery of the students on their way to school
	Conservation flagship	The black-necked crane coexists peacefully with people and promotes the harmonious development of the relationship between people and animals
		Because of the Black-necked crane, everyone living near Caohai has the consciousness of protecting Caohai
		It is a treasure given to human beings by nature and a good friend of human beings
		It helps people better reflect on the relationship between people, animals, and nature
		It does not only strengthen people’s awareness of bird conservation but also strengthens people’s awareness of protecting wild animals, biodiversity, and ecosystems
	Bequest value	We should protect the crane so future generations can better understand the bird and also pass on the sense of conservation and tradition to the next generation, ensuring the black-necked crane can exist in the real world forever
	Traditional knowledge	When it comes, it reminds farmers that they can harvest their crops, and when they leave, they remind them that they can sow crops
		The coming and going of the black-necked crane tells us that the year is coming to an end and the following year is coming
		It provides human beings with the advantage of better discerning direction
		The song of the black-necked crane in the morning is sometimes like an alarm clock and will wake people up from their sleep to start a new day
	Formal knowledge	It has excellent research value in the fields of biology and ecology
	Education	Weining children can observe wild animals more closely, learn more about birds, and broaden their horizons
		The black-necked crane is constantly migrating many times of the year, and the persistent spirit of migratory birds from north to south tells us that if we want to succeed, we need to stick to it
		The black-necked crane eats rotten crops (bad potatoes and corn) in the fields and food that people drop in the field, which makes people realize that food should not be wasted

### Conservation attitude

Among the students, 37.52% (n = 391) listed the black-necked crane and/or the Caohai wetland as among the top-three famous things in the region, indicating that the bird and the wetland have a position in their minds. Accordingly, 80.81% (n = 842) of the students agreed that the bird should be conserved. Conservation attitudes were mainly determined by awareness (Table 6, *β* = 1.55; P < 0.001), knowledge (*β* = 0.99; P < 0.001), and grade (*β* = 0.46; P < 0.05). Students who knew the black-necked crane have a 4.95 times higher conservation attitude than those who did not (Exp.coef = 4.95). The odds ratio between the 9th and 7th grade students is 1.39 (Exp.coef = 1.39), while the odds ratio for knowledge is 2.86 (Exp.coef = 2.86).

**Table 6:** Logistic regression analyses of factors that significantly impacted students’ conservation attitude toward the black-necked crane

	B	SE	z value	Pr ( >|z|)
Intercept	− 3.72	1.63	− 2.28	0.02
School	− 0.03	0.05	− 0.78	0.44
Grade	0.46	0.21	2.23	0.03
Age	− 0.11	0.16	− 0.68	0.50
Gender	− 0.19	0.25	− 0.76	0.45
Ethnicity	− 0.57	0.36	− 1.60	0.11
Address	− 0.16	0.16	− 1.01	0.32
Observation	0.39	0.34	1.15	0.25
Visiting experience	− 0.12	0.29	− 0.41	0.68
Awareness	1.55	0.41	3.79	0.0002
Knowledge	0.99	0.13	7.54	1.05e−13

## Discussion

### Knowledge and formal education impact

Although this study showed that most students have positive attitudes toward the conservation of the black-necked crane, they have limited knowledge of the birds’ biology and services. Most students knew that it is a species of bird and winters in the Caohai wetland but failed to demonstrate an in-depth understanding of the black-necked crane, such as its conservation status, diet composition, functional roles in the ecosystem, and the associated services. For example, only 76.87% of students know it is a first-level national key protected wild animal in China despite that the Caohai wetland has been set up as a protected area for more than 30 years. A few students even thought it does not eat crops but pest insects (8 urban and 18 rural students, respectively). This is highly unlikely since insects are almost absent or inactive in winter in this place. Similarly, only 19.10% of students recognized that the black-necked crane is an important tourism resource in Weining.

Our analyses showed that the students’ conservation attitudes were positively related to their level of knowledge. Therefore, correcting the students’ misconceptions and imparting general biological knowledge and values of the black-necked crane will likely move these young pupils toward becoming more knowledgeable, bird-friendly adolescents and eventually adults willing to positively anticipate conservation activities. Furthermore, a strong positive correlation between attitudes and knowledge has been reported in several other studies, even for the non-charismatic species. For example, people with more knowledge showed a more positive attitude toward bats [18].

Our study showed that formal education in school has contributed to the students’ knowledge of biology and the value of the black-necked crane. Students in Grade 9 have a higher average score than those in Grade 7. This difference can be attributed to the school curriculum of biology offered in this region. Biology in Grade 7 is focused on plants and animals in Grade 8. At the time of this survey, students in Grade 7 had little knowledge of animals and plants since this was the first semester. In contrast, students in Grade 9 have completed the whole biology curriculum. This indicated that even general zoological knowledge could benefit the students’ attitude toward the black-necked crane. Additionally, general zoology knowledge may have inspired their interests in animals, encouraging them to seek further knowledge of the black-necked crane from other available sources, such as social media reports by other students. Furthermore, students in Grade 9 reported having more experiences with the Caohai wetland than those in Grade 7, which may reflect their eagerness to watch the black-necked crane.

However, our survey showed that social media is the most cited source to acquire knowledge of the black-necked crane. This indicated that more efforts could be made to disseminate information on the black-necked crane in schools. For example, conservation experts in and out of the local conservation agency could give the students lectures on the black-necked crane, focusing on a detailed introduction to its biology, ecology, conservation, and ecosystem services. These experts could also share handouts, photographs, and videos of the bird with schools, which can foster learning and awareness education in the teachers, especially for schools far away from the Caohai wetland ecosystem.

### Carrying out education in nature

While learning is an active and individual construction of knowledge, it is intimately connected with what people experience [43]. Increasing the number of direct experiences can be an effective strategy to improve students’ level of wildlife knowledge, conservation awareness, and willingness to participate in conservation activities [44]. For example, a ten-week family-based wild bird feeding education program showed that children had a significant increase in bird knowledge, and 90% of the contacted families were still feeding birds one year later [45]. Furthermore, experiences with the natural environment can encourage students to understand their living environments, which are the precondition for them to develop a responsible attitude toward the environment [46]. Some scholars even connected the loss of direct experiences in the natural environment with the global loss of biodiversity. Therefore, an essential step toward a sustainable future is to prevent the loss of direct experience [47]. In this regard, education in nature could be the best way to prevent the loss of direct experience. Students need regular contact with the natural environment in a school context, especially those that do not have access to nature as part of their everyday lives [48].

Our data also showed that the students’ knowledge was significantly influenced by the opportunities for direct observation of the bird. This indicated that improving their knowledge level and shaping their favorable attitudes could be achieved by increasing the direct experiences students have with the crane. Notably, the students whose family residency is far from Caohai have little chance to visit the Caohai wetland and enjoy direct contact and experience with the black-necked crane. Therefore, it is suggested that these schools could carry out biological education in the Caohai wetland one or two times per year through field trips and activities, such as bird-watching and bird feeding, guided by the local conservation experts and other conservation initiatives. However, this requires that the local education governmental agency provides specific or sufficient funding for such educational activities.

### Incorporating local ecological knowledge into education

Local ecological knowledge has been widely considered a valuable tool to help conserve and maintain biodiversity in the past decades. This kind of knowledge is built on the local population’s accumulation of observations and experiences in a specific surrounding. To a certain degree, it reflects the intimate interactions between local people and the ecosystem supporting their living, contributing to a culture integrated with nature [49, 50]. One of the unique characteristics of the local ecological knowledge is that it was orally transmitted between generations within the communities. Parents, family relatives, and friends can make instrumental contributions to the persistence of the local ecological knowledge.

Due to the habitat selection for resting and foraging, the black-necked crane has closely interacted with the local populations around the Caohai wetland, especially the farmers and fishers. The intimate contact has contributed to the development of local ecological knowledge, which has been passed down to the current generation. This study showed that the bird has infiltrated into local people’s daily lives with a high cultural significance (defined as the value and role of a species in human cultures). For example, as mentioned by a few students, important farming activities, such as sowing and harvesting, are carried out according to the regular migration time of the bird, and the time to get up can be alarmed by the bird’s morning vocalizations. Most importantly, the locals have embedded the bird into moral and character education, such as the key to success and the need to save food (Table 5). Obviously, this traditional knowledge and relevant culture can flag a positive image of the black-necked crane for the students and should be incorporated into the conservation awareness program.

## Conclusions

This study found that most students have a positive attitude toward the conservation of the black-necked crane, and the knowledge they own is a significant determining factor. Unfortunately, most students have limited knowledge of the birds’ biology and ecosystem services. Although several variables impact knowledge, it is predominantly determined by direct contact with the bird. Therefore, the students with a higher score on knowledge usually have more direct experiences with the bird. These findings have significant implications for raising students’ conservation awareness on the black-necked crane. It is vital to shape more positive attitudes toward the bird and its conservation, especially in those students whose residences are far away from the Caohai ecosystem. To accomplish this, students should be provided with more in-depth scientific and local knowledge, and the distance between the students and the bird should be shortened by carrying out education activities in nature.

In summary, one key group that should be considered to ensure the success of conservation efforts is today’s adolescents, as they are the future decision-makers and practitioners [51]. A few empirical studies have shown that wildlife education can enhance pupils’ knowledge and change their awareness and attitudes toward conservation [52]. Improving students’ knowledge of the black-necked crane may encourage more positive attitudes and motivate positive conservation behaviors. To our knowledge, this is the first study focused on the human dimension for the conservation of the black-necked crane in China. The implications presented in this study highlighted the urgent need to carry out similar studies in other regions where this bird or other protected species is distributed. However, one limitation of this study is that it only focused on secondary students. Therefore, future assessments should be conducted among the other local populations, such as farmers, fishers, and urban citizens. This could enable the design and application of effective measurements to improve local people’s perceptions and attitudes toward conserving the black-necked crane.

## Data Availability

The datasets used and/or analyzed during the current study are available from the corresponding author on reasonable request.
